# Mind Wandering and Task-Focused Attention: ERP Correlates

**DOI:** 10.1038/s41598-018-26028-w

**Published:** 2018-05-15

**Authors:** Óscar F. Gonçalves, Gabriel Rêgo, Tatiana Conde, Jorge Leite, Sandra Carvalho, Olívia Morgan Lapenta, Paulo S. Boggio

**Affiliations:** 1 Psychological Neuroscience Laboratory– CIPsi, School of Psychology, University of Minho, Braga, Portugal; 2 Spaulding Neuromodulation Center, Department of Physical Medicine & Rehabilitation, Spaulding Rehabilitation Hospital and Massachusetts General Hospital. Harvard Medical School, Boston, USA; 3 Social and Cognitive Neuroscience Laboratory, Center for Health and Biological Sciences, Mackenzie Presbyterian University, São Paulo, Brazil; 4 Faculdade de Psicologia, Universidade de Lisboa, Lisbon, Portugal; 5 Portucalense Institute for Human Development (INPP), Universidade Portucalense, Porto, Portugal; 6 The MARCS Institute for Brain, Behaviour & Development, Western Sydney University, Penrith, Australia

## Abstract

Previous studies looking at how Mind Wandering (MW) impacts performance in distinct Focused Attention (FA) systems, using the Attention Network Task (ANT), showed that the presence of pure MW thoughts did not impact the overall performance of ANT (alert, orienting and conflict) performance. However, it still remains unclear if the lack of interference of MW in the ANT, reported at the behavioral level, has a neurophysiological correspondence. We hypothesize that a distinct cortical processing may be required to meet attentional demands during MW. The objective of the present study was to test if, given similar levels of ANT performance, individuals predominantly focusing on MW or FA show distinct cortical processing. Thirty-three healthy participants underwent an EEG high-density acquisition while they were performing the ANT. MW was assessed following the ANT using an adapted version of the Resting State Questionnaire (ReSQ). The following ERP’s were analyzed: pN1, pP1, P1, N1, pN, and P3. At the behavioral level, participants were slower and less accurate when responding to incongruent than to congruent targets (conflict effect), benefiting from the presentation of the double (alerting effect) and spatial (orienting effect) cues. Consistent with the behavioral data, ERP’s waves were discriminative of distinct attentional effects. However, these results remained true irrespective of the MW condition, suggesting that MW imposed no additional cortical demand in alert, orienting, and conflict attention tasks.

## Introduction

Compared to its small contribution to total body mass (~2%), the human brain contributes to a large proportion of the body’s overall energy consumption (20%). A large majority of the brain’s metabolism is associated with spontaneous activity (70–80%), with task evoked activity accounting for for only 5% of the energy consumption^1^. Estimates suggest that most of this spontaneous brain activity is associated with self-generated thoughts rather than external and task-related activity. This condition, known as mind wandering (MW), occupies most of our daily lives^2^, and may have important adaptive functions. Furthermore, there is now extensive evidence that suggests that MW plays an important role in processes such as autobiographic planning^3^ and creativity^3^.

Despite the beneficial effects on creativity and daily planning, the perceptual decoupling that occurs during MW is thought to have significant attentional costs^4^. Interference between MW and attention may be due to the use of overlapping executive resources required in MW and attention^5–7^. This is further confirmed by brain imaging studies showing that MW tends to recruit not only the Default Mode Network, but also brain networks associated with executive functioning^8^.

However, there is increasing evidence that MW does not affect all types of attention tasks^7^. Experiments on the relationship between attention and MW have been producing inconsistent results^9,10^. These conflicting results may be explained by different concepts and methods used to assess both MW^11^ and attention^12^. Some studies, define MW as an over inclusive category referring to all type of task-unrelated thoughts^7^. Contrarily, other studies provide a less inclusive definition that restricts MW to stimuli independent and task unrelated thoughts (e.g., thoughts dissociated either from the task or the current external conditions such as wandering about past memories or future plans) and excludes task related (e.g., thoughts on side aspects of the task such as task duration, concerns about overall performance, rumination over a mistake) or external (e.g., thoughts about task unrelated external and internal stimuli, such as light, temperature, hunger, thirst) distractors^13^.

The multiple approaches to assess MW can be divided in two major strategies: real-time and retrospective reports^14^. In real-time strategies, a thought-probe interrupts the task and prompts participants to report their ongoing conscious experience. These real-time strategies seem to be a reliable method for MW assessment, however they tend to interrupt the ongoing task^15^. To overcome this limitation, some studies use a retrospective strategy, collecting participants’ reports after the task by using either structured interviews or questionnaires^16^. This strategy, even though more prone to reliability issues, has the advantage of not interfering with the ongoing task. A good example of a retrospective strategy is the Resting State Questionnaire (ReSQ)^17^ in which participants are requested to report the percentage of time spent on visual mental imagery, inner language, somatosensory, inner musical experience, and mental manipulation of numbers. Previous studies show that a significant percentage of individuals could be classified in a dominant MW mode^17^ and that ReSQ is an effective method for assessing MW in resting state studies^18^.

Recently, MW research has evolved to use various experimental paradigms to approach the assessment of attention^15,19^. Acknowledging that attention is not a single mechanism led researchers to move away from the use of single sustained attention tasks and towards composite attention measures, such as the Attention Network Task (ANT)^20,21^. ANT is a computerized visual–motor task designed to assess three attentional networks: alerting, orienting, and conflict^22,23^. Alerting is defined as the process of reaching and maintaining a state of responsiveness to external stimuli and is assessed by looking at the facilitative effect of non-spatial informative cues (double cues) compared with the no cue condition. Orienting refers to the ability to select among multiple stimuli and is assessed by the facilitative effect of spatial informative cues compared with non-spatial informative cues. Finally, conflict refers to the executive monitoring of performance requiring inhibitory control, and is assessed by measuring the interference effect of incongruent flankers compared to congruent flankers.

At the behavioral level, studies looking at how MW interferes with the performance of distinct FA systems using the ANT^10,16^, showed that MW (i.e., defined as stimulus independent and task unrelated thoughts) did not impact overall performance and efficiency on each network assessed by the ANT (alert, orienting and conflict). One explanation for these results is that, while MW and focused attention thoughts (FA) share executive brain network resources, MW additionally recruits default mode network (DMN) resources^24^. The DMN is a networking connecting the medial frontal cortex with the posterior cingulate, precuneus and inferior parietal cortex. These brain regions show a higher activation during the rest condition and are usually deactivated when the individual performs a task requiring directing attention to an external stimulus^25^. Recent studies provided evidence that DMN may play an important role in high-level social cognitive processes such as self-referential thought^26^ and social processing^27^.

Similar performances can be achieved by compensating executive losses with complementary brain network recruitment. Therefore, it is possible that MW may not only compete but also facilitate attention processes, such as attention recycling, dis-habituation, and mood regulation^28^. Noteworthy, several studies confirmed that increased functional connectivity in resting state networks (e.g., DMN) is associated with the properties of different event related potentials (ERP’s) proprieties^29^. Therefore it would be of interest to explore how MW modulates the electroencephalographic potentials found to be markers of the ANT.

Several neurophysiological studies have been looking at the ERP correlates associated with different ANT networks. For example, Neuhaus and colleagues^30^ reported an increase in N1 amplitude in response to target onset following alert and orienting cues, thus suggesting that, at sensory stages of visual information processing, attentional processes underlying alerting and orienting networks operate concurrently. Moreover, they observed a frontal P3 increase and parietal P3 decrease for incongruent targets, pointing to a distinct topographic modulation of P3 amplitude by response inhibition. Later, Galvao-Carmona and colleagues^31^ showed increased CNV amplitude to spatial cues before target onset, when compared with central and no cue conditions. They also found an increase amplitude in P1 for the spatial cue and increase in N1 components for the central cue after the target onset, and P3 amplitude for the congruent compared with incongruent targets. More recently, Williams and colleagues^32^ also reported also an increase in N1 and CNV amplitude before the target presentation (as well as after the target for N1) for the double cue when compared with the no cue (alerting effect) and the spatial cue when compared with the central (orienting effect). Additionally, they found an increase in P1 after the target for the no cue condition, when compared with the double cue (alerting effect); and for the central cue, when compared with the spatial cue (orienting effect). The authors also reported an increase in N2 and a decrease of P3 amplitudes for incongruent targets. Kaufman and colleagues^33^ confirmed the increase in N1 in response to the double cue when compared with the no cue (alerting effect) and to the spatial cue when contrasted with the center cue (orienting effect) after target presentation (particularly in young adults) along with a reduced P3 amplitude for incongruent targets (conflict effect).

Even though we are not aware of any studies looking into how MW impacts ANT performance as assessed by EEG, several studies using other attention paradigms suggest that MW affects several ERP components in FA tasks. For example, using a go/no-go task, Smallwood and colleagues^34^ reported a decrease in P3 amplitude for no targets every time participants start mind wandering. In an oddball paradigm, Braboszcz and Delorme^35^ found an increase in P2 amplitude during mind wandering for both oddball and standard auditory stimuli. Finally, Baird and colleagues^36^ reported an attenuation of the P1 amplitude during mind wandering during a 0-back vigilance task.

Altogether, cue-locked CNV, N1 and target-locked N1 and P1 components seem to represent reliable ERP signatures of the alerting and orienting networks, while the target-locked P3 is a good marker for the conflict network. Studies using other experimental paradigms (e.g., go/no-go, odd-ball, vigilance task) found some of these components (e.g., P1, P3) to be also impacted by MW. Recently, several studies claimed the existence of prefrontal activity associated with alert or discriminative attention processes in distinct paradigms (e.g., pN, pP1)^37^. Research shows that those brain waves may be sensitive to a compensatory prefrontal load to maintain optimal performance in face of individual (e.g., aging) or situational constrains (e.g., conflict)^38,39^.

Building on these findings we hypothesize that a distinct cortical processing may be required to meet increasing attentional demands during MW. We tested if, given similar levels of ANT performance, individuals predominantly focusing on MW or FA show distinct neurophysiological processing, as evidenced by distinct properties of the following ERP’s during ANT: pN1, N1, pP1, P1, pN (cue -locked), and pN1, N1 pP1, P1, P3 (target-locked).

## Method

### Participants

Thirty-three healthy individuals (22 female) with normal or corrected-to-normal vision took part in this study. Ages ranged from 18 to 40 years, with a mean of 23.45 years (SD = 5.01 years). Inclusion criteria were: (i) right handedness^39^; (ii) no metal implants on the head; (iii) no history of neurological or psychiatric illness, electroconvulsive treatment, drug or alcohol abuse in the past year; (iv) no current medication for medical disorders that would impact electroencephalogram (EEG) morphology; (v) a score in Beck Depression Inventory (BDI)^40^ ≤18. After a detailed description of the study, all participants provided signed informed consent, and the study was carried out under The Code of Ethics of the World Medical Association (Declaration of Helsinki). The study was approved by the Institutional Review Board of the Mackenzie Presbyterian University and by the National Ethics Committee (SISNEP, Brazil), and all participants had provided written informed consent.

### Materials

#### Attention network test (ANT)

The ANT is a computerized task, designed to assess three attentional networks: alert, orienting, and conflict. Participants are required to focus on a central fixation cross and identify if the target (i.e., central arrow appearing below or above a fixation cross) is pointing right or left. The targets are preceded by three cue conditions: a spatially informative cue presented above or below the fixation cross, a center or double time informative cue, and a no cue condition. The target may be presented alone or accompanied by three distinct flankers: arrows pointing in the same direction of the target (congruent condition), pointing in an opposing direction (incongruent condition), or traces without arrows (neutral condition).

**Figure 1:**
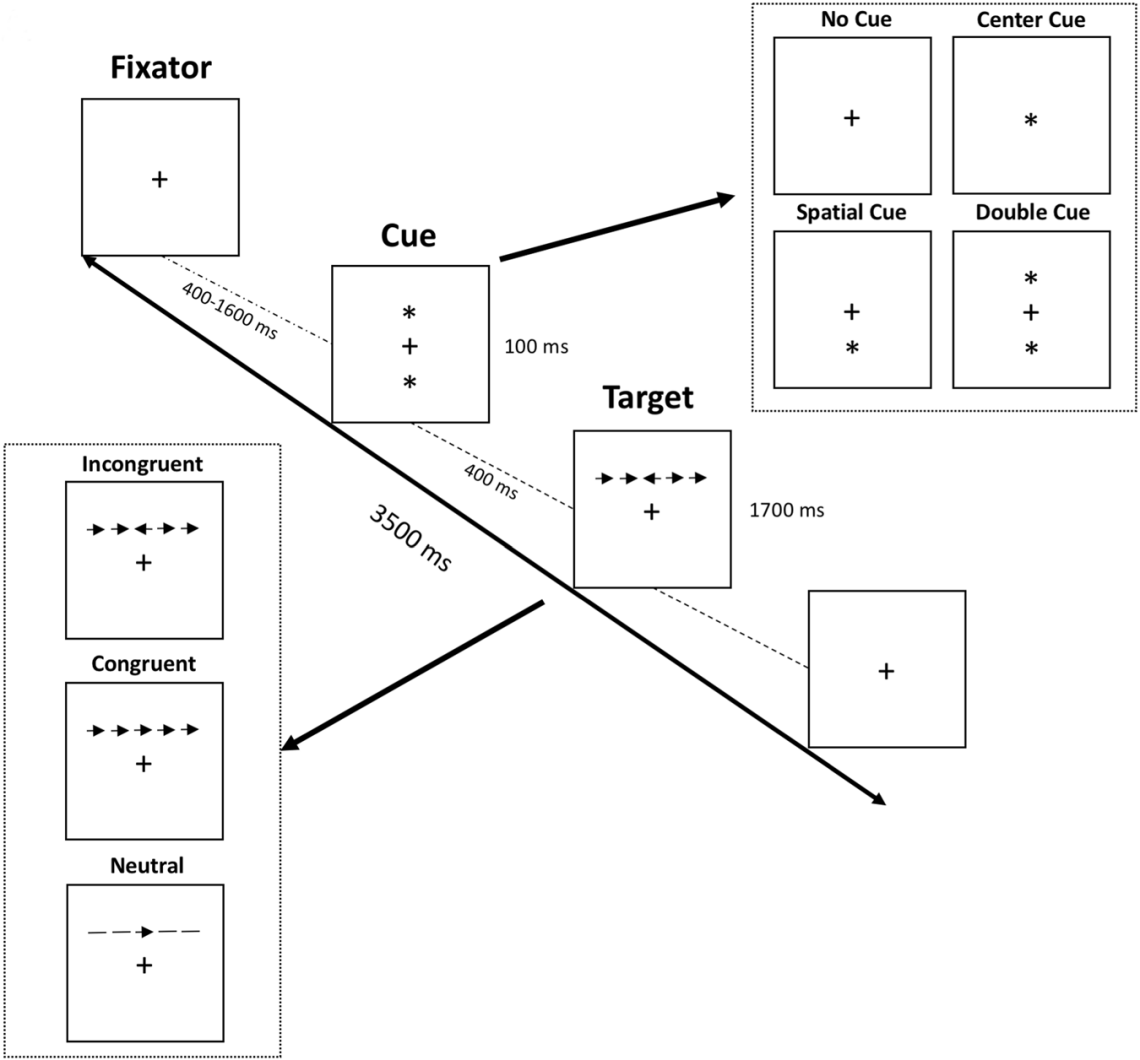
Attention Network Task - Experimental paradigm.

ANT was programed and presented via E-Prime 2.10 (Psychology Software tools, Sharpsburg, PA, US) in a desktop computer, using the following parameters: (1) a fixation cross appeared and remained in the center of the screen throughout the entire trial; (2) after a random duration varying between 400 and 1600 ms, a cue (none, center, double or spatial cue) appeared for 100 ms; (3) after a fixed period of 400 ms, the target (the center arrow) and flankers (congruent, incongruent or neutral flankers) were presented until the participant responded but with a time limit of 1700 ms (participant’s response was done by pressing either the right or the left side of the computer mouse with their dominant hand); (4) after the response, the target and flankers were replaced by the central fixation cross (the time lapse between the onset of the target and the start time of the next trial comprised 3500 ms *minus* both the participant’s reaction time and the duration of the first fixation cross). A session consisted of seven blocks: one full-feedback practice block and six experimental blocks without feedback. Each experimental block included 48 trials (4 cue conditions × 2 target locations × 3 flanker conditions × 2 repetitions). In every block, trials were presented in a random order (see Fig. 1).

The ANT allows the computation of three attentional systems: alerting, orienting and conflict. In the present study, the alerting network is defined by the facilitative effect of the double cue compared with the no cue condition. The orienting network refers to the facilitative effect of the spatial informative cue (above and below the fixation cross) compared with central cue. The conflict network expresses the interfering effect of incongruent flankers compared with congruent flankers.

Even though researchers are still searching for more reliable methods of computing ANT network effects^41,42^, studies show that these three attentional components are supported by different neuroanatomical networks^22^ and are associated with distinct genetic profiles^43^.

#### Mind Wandering Task (MW)

Upon ANT completion, all participants were asked to fill out an adapted version of the Resting State Questionnaire (ReSQ)^17^ and report the percentage of time spent in the following categories of mental activity: focusing on the task (FT); visual mental imagery - seeing something in thought (IMAG); inner language - thinking in words with your own voice without overt production (LANG); somatosensory awareness - paying attention to a sensory aspect of the body (SOMA); inner musical experience - experiencing a melody and/or a rhythm in thought (MUSI); and mental processing of numbers - arithmetic processing, counting, estimation of the time to the end (NUMB). The sample was divided in two groups based on their responses to the ReSQ using a modified version of the half split method. Participants that report to have been over 60% focused on task were classified as the “focused attention” (FA) group (n = 13), while those reporting over 60% in the remaining conditions were classified in the “mind wandering” (MW) group (n = 20). Participants were excluded from further analysis if they did not reach a score of 60% in one of the categories (in the current study all participants were included). MW and FA groups did not differ in terms of sex (Χ^2^ = 0.06, p = 0.80), age [*t*(31) = 0.07, *p* = 0.93] and years of education [*t*(31) = 1.26, *p* = 0.22]. The average percentages for each ReSQ category were: FT–45.76%; IMAG–14.32%; LANG–18.53%; SOMA–7.71; MUSI–11.29%; NUMB–2.09%.

### Procedure

After providing signed informed consent and before the experimental trials, participants went through the following steps: (1) instructions about the overall procedure; (2) a practice block of the ANT with feedback; (3) six ANT experimental blocks synchronized with EEG acquisition (see Fig. 1); (4) Completing the ReSQ.

### EEG data acquisition and analysis

A high-density 128 geodesic sensor net (Electrical Geodesic) was used for EEG data acquisition. Impedances were kept below 40 kΩ at the beginning of the experiment, and monitored throughout the entire session. EEG was sampled at 250 Hz, band-pass filtered between 0.01–100 Hz (Notch filter at 60 Hz), and stored for later analysis. EEG recordings were referenced to Cz and later referenced offline to the average-reference. The following steps were used for EEG processing: (1) ERPs for correct trials were segmented into epochs that were time-locked according to cue onset and target onset (100 ms before and 600 ms after either the cue or target onset); (2) artifact detection (difference >140 μV between channels above and below the eyes, difference >55 μV between channels near the outer canthi, or one or more channels exceeding an amplitude of 200 μV); (3) re-referencing of scalp potentials to the average reference; (4) baseline correction from 100 ms before each segment. Epochs containing artifacts due to eye blinks, ocular and head movements were automatically rejected.

**Figure 2:**
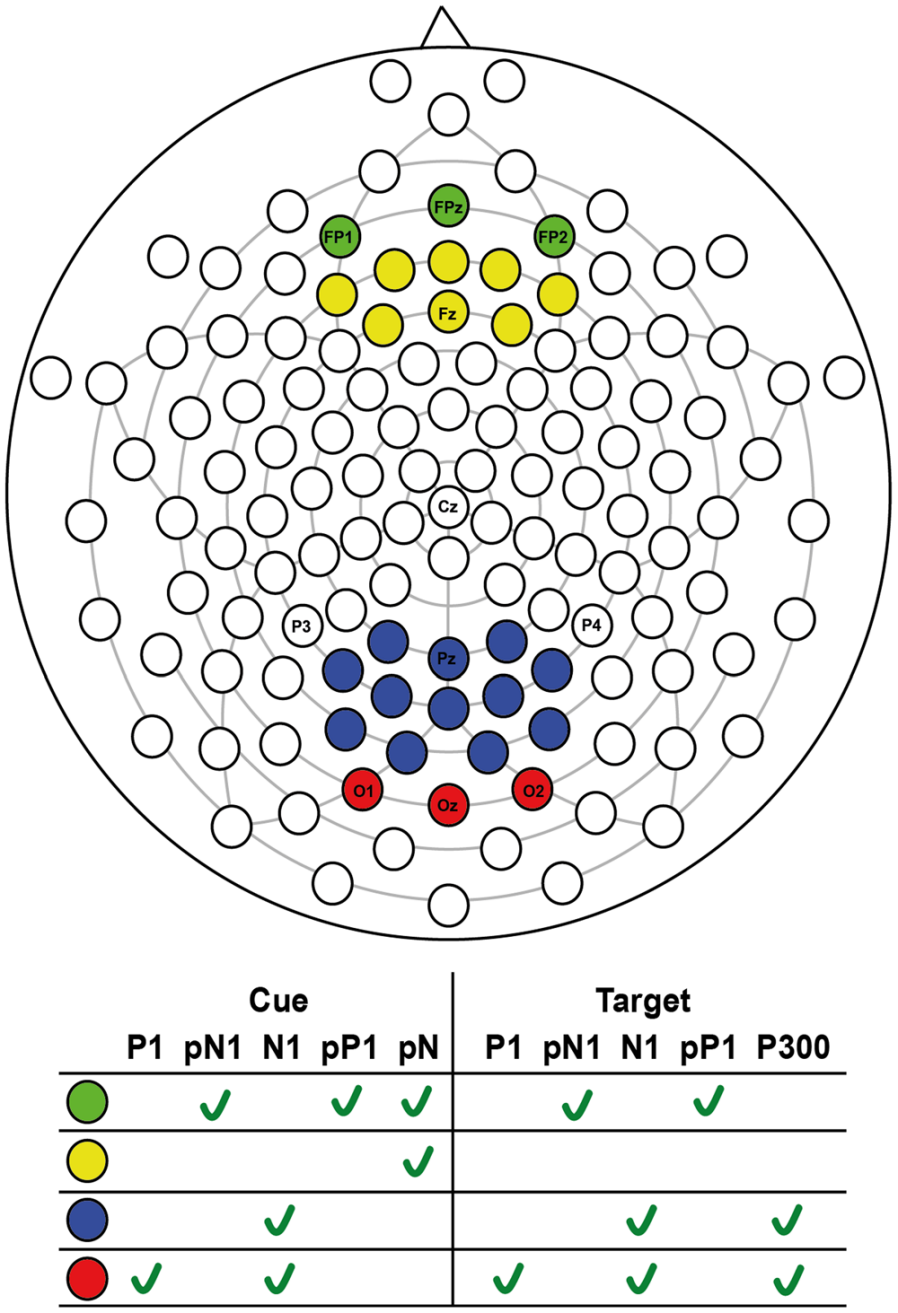
Scalp sites for cue and target locked ERP’s.

After visually inspecting the grand average ERP waveforms, cue-locked P1, pN1, N1, pP1 and pN and the target-locked, P1, pN1, N1, pP1 and P3 ERP components were selected for further analyses. Electrode sites shown in Fig. 2 were chosen according to previous research. Adaptive mean amplitudes were analyzed within the 90–150 ms time window after cue and/or target for P1 (at O1, O2, and OZ) and for pN1 (FP1, FP2, FPz), 150–250 ms for pP1 (at FP1, FP2, FPz), 160–260 ms for N1 (at O1, O2, and OZ and Pz and adjacent electrodes), and 300–450 ms for P3 (at O1, O2, and OZ and Pz and adjacent electrodes). A slow negative potential (i.e., CNV like) was most prominent in prefrontal (FP1, FP2, FPz) and frontal scalp regions (Fz and adjacent electrodes) between 350–500 ms after cue onset. Due to its topography, we will refer to this type of wave, now on, as prefrontal negativity (pN).

In every condition, at least 70% of artifact-free segments were included in the individual ERP averages (No Cue = 89.56 ± 7.32; Center Cue = 87.88 ± 8.45; Double Cue = 87.75 ± 9.18; Spatial Cue = 86.40 ± 9.99; Congruent = 88.98 ± 7.28; Incongruent = 87.15 ± 9.06; Neutral = 87.56 ± 9.20). The groups did not differ in terms of the number of artifact-free epochs included in the individuals grand-averages per condition [*F*(1,192) = 1.42, *p* = 0.21].

### Data availability

The datasets generated during the current study are available from the corresponding author on reasonable request.

## Results

### Relationship Between Focused Attention and Mind Wandering –Behavioral Results

**Figure 3:**
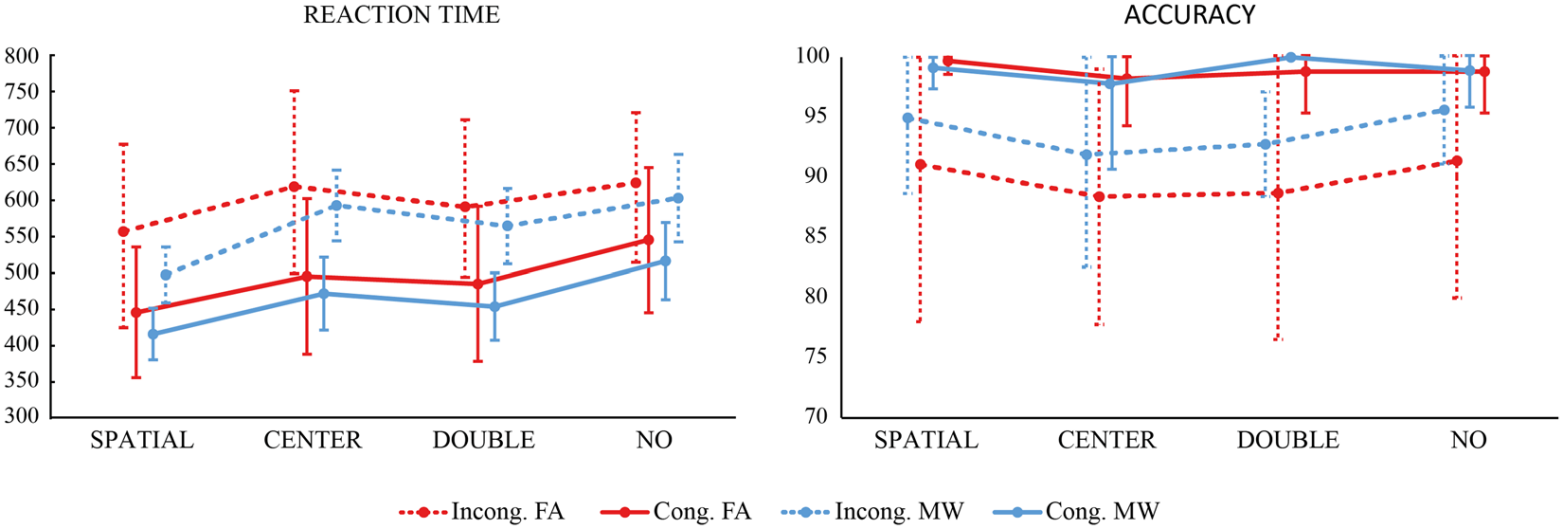
ANT Reaction times (**a**) and accuracy (**b**) in the FA and MW groups for different cue and target conditions (mean ± SD).

For the analyzes of behavioral results, two repeated-measures analysis of variance (ANOVAs) were performed with ANT’s reaction time (RT) or accuracy score as dependent variables and type of cue (double cue, center cue, no cue and spatial cue), type of target (congruent, neutral, incongruent) as the within-subject factors, and mind-wandering (MW, FA) as between-subjects factor. For RT there was a significant main effects for target [*F*(1,31) = 503.31, *p* < 0.001, *ηp*^2^ = 0.94] and cue conditions [*F*(3,93) = 128.49, *p* < 0.001, *ηp*^2^ = 0.81] as well as cue*target interaction [*F*(3,93) = 10.23, *p* < 0.001, *ηp*^2^ = 0.25]. No significant effects were found for mind-wandering [*F*(1,31) = 1.34, *p* < 0.26, *ηp*^2^ = 0.04] and for the interactions between cue*mind-wandering [*F*(3,93) = 0.37, *p* < 0.77, *ηp*^2^ = 0.01], target*mind-wandering [*F*(1,31) = 0.16, *p* < 0.69, *ηp*^2^ = 0.01] and cue*target*mind-wandering [*F*(3,93) = 1.43, *p* < 0.24, *ηp*^2^ = 0.04]. Bonferroni post-hoc tests showed significant differences between all conditions (*p* < 0.001) except for the following conditions (*p* > 0.05): double-congruent and center-congruent; no cue-congruent and spatial-incongruent; and between no cue-incongruent and center incongruent (see Fig. 3).

In terms of accuracy, significant main effects were found for target [*F*(1,31) = 39.47, *p* < 0.001, *ηp*^2^ = 0.56] and cue conditions [*F*(3,93) = 3.06, *p* = 0.03, *ηp*^2^ = 0.09], but not for the cue*target interaction [*F*(3,93) = 1.98, *p* = 0.12, *ηp*^2^ = 0.06]. Again, no significant effects were found, for mind-wandering [*F*(1,31) = 1.81, *p* = 0.19, *ηp*^2^ = 0.06] or for the interactions between cue*mind-wandering [*F*(3,93) = 0.55, *p* = 0.64, *ηp*^2^ = 0.02], target*mind-wandering [*F*(1,31) = 2.67, *p* = 0.11, *ηp*^2^ = 0.08] and cue*target*mind-wandering [*F*(3,93) = 0.18, *p* = 0.91, *ηp*^2^ = 0.01]. Bonferroni post-hoc tests showed that the only significant difference was between center and no cue conditions (*p* = 0.05).

### Relationship Between Focused Attention and Mind Wandering –ERP Results

**Figure 4:**
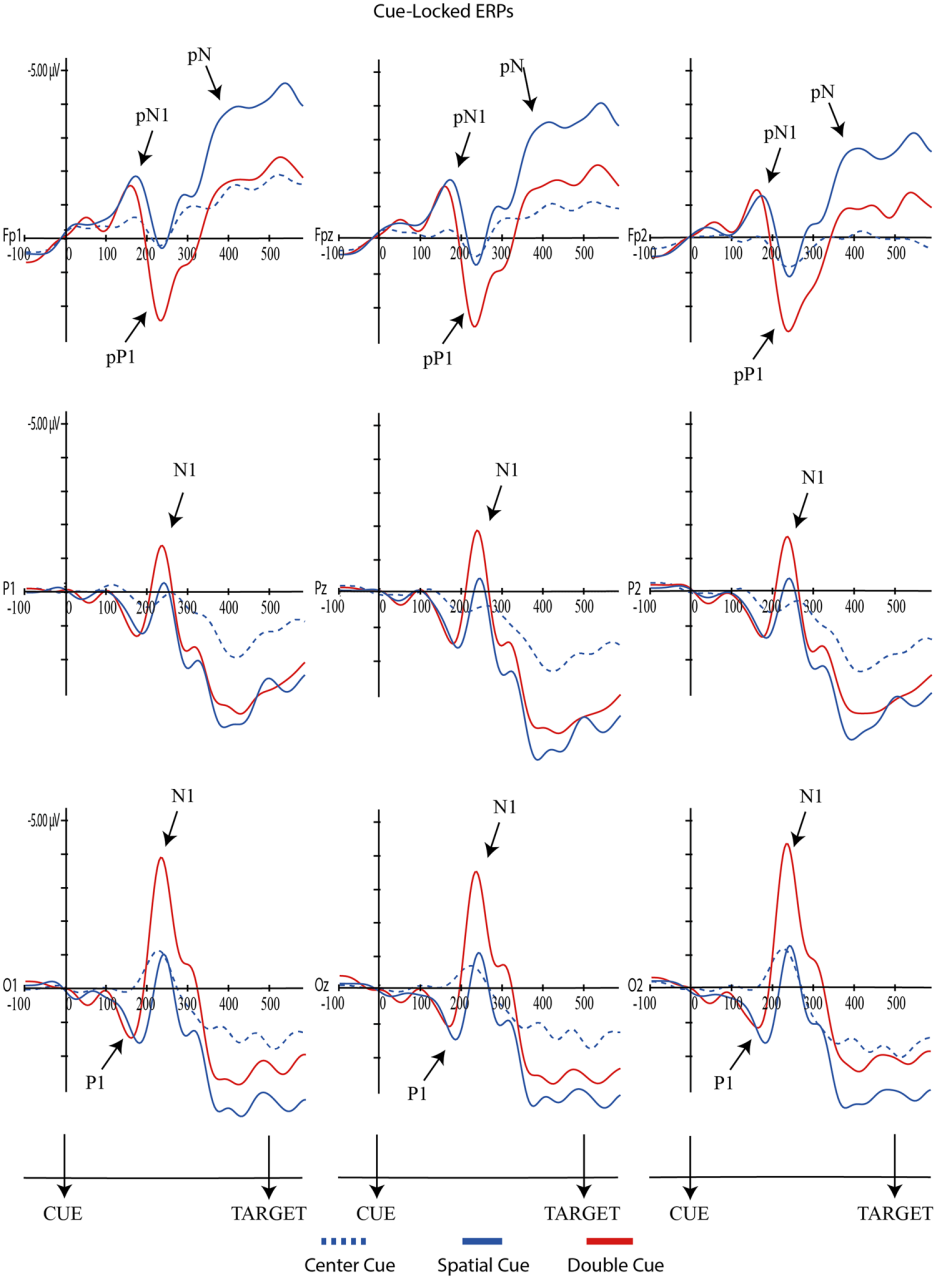
Grand averages for cue locked ERP components.

**Figure 5:**
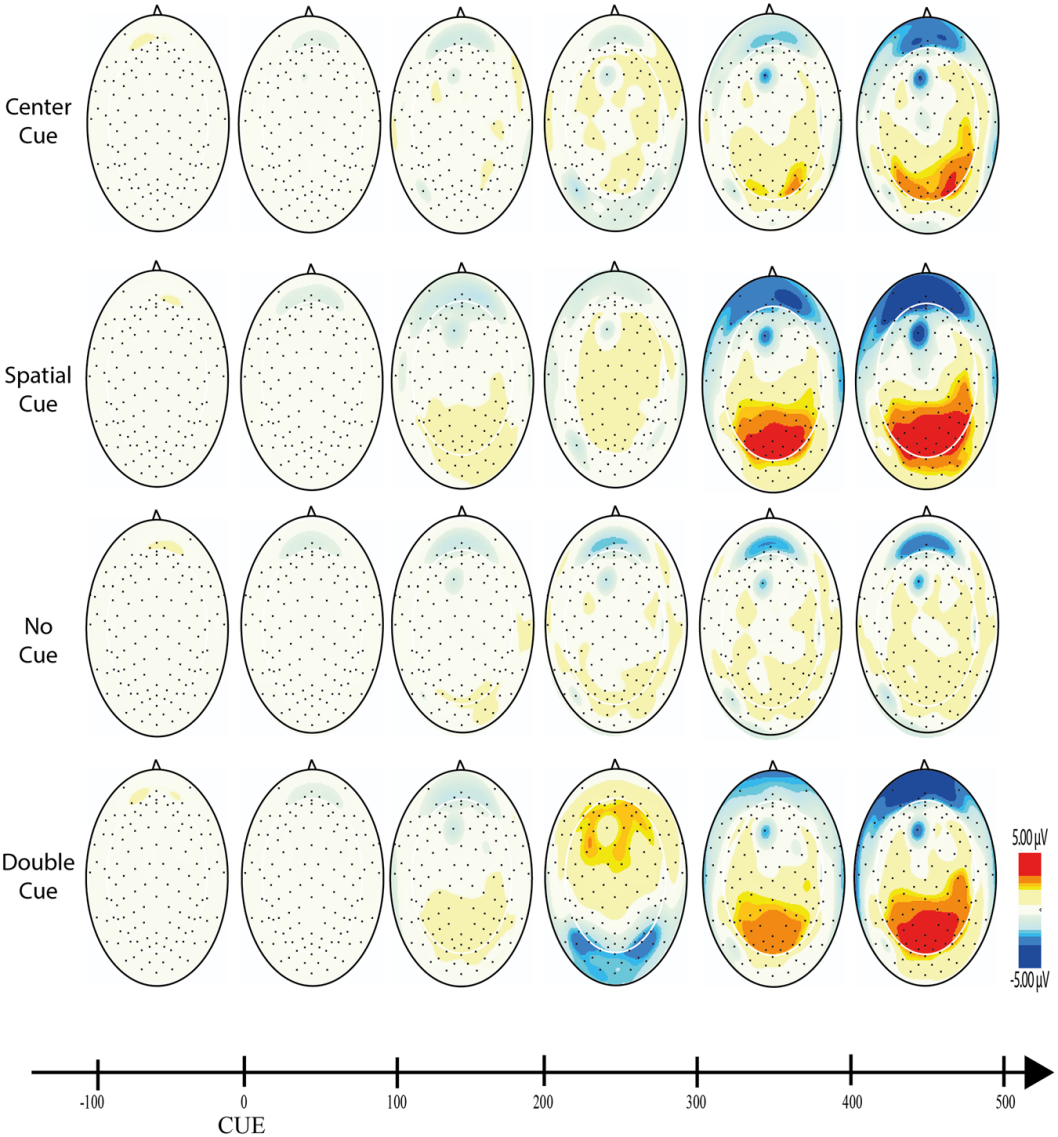
Topographical maps for cue locked ERP components.

**Figure 6:**
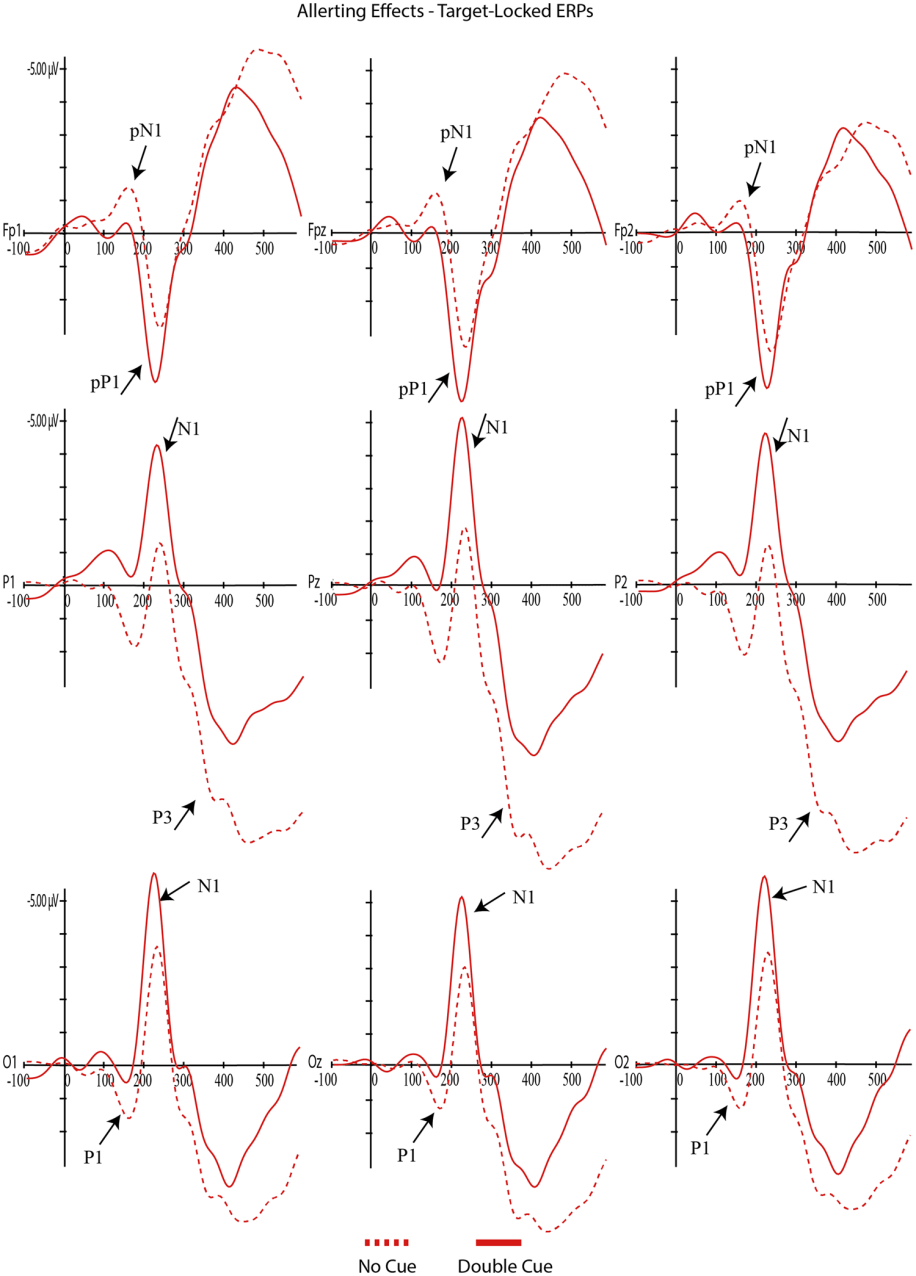
Grand averages for target locked ERP components – alerting effects.

**Figure 7:**
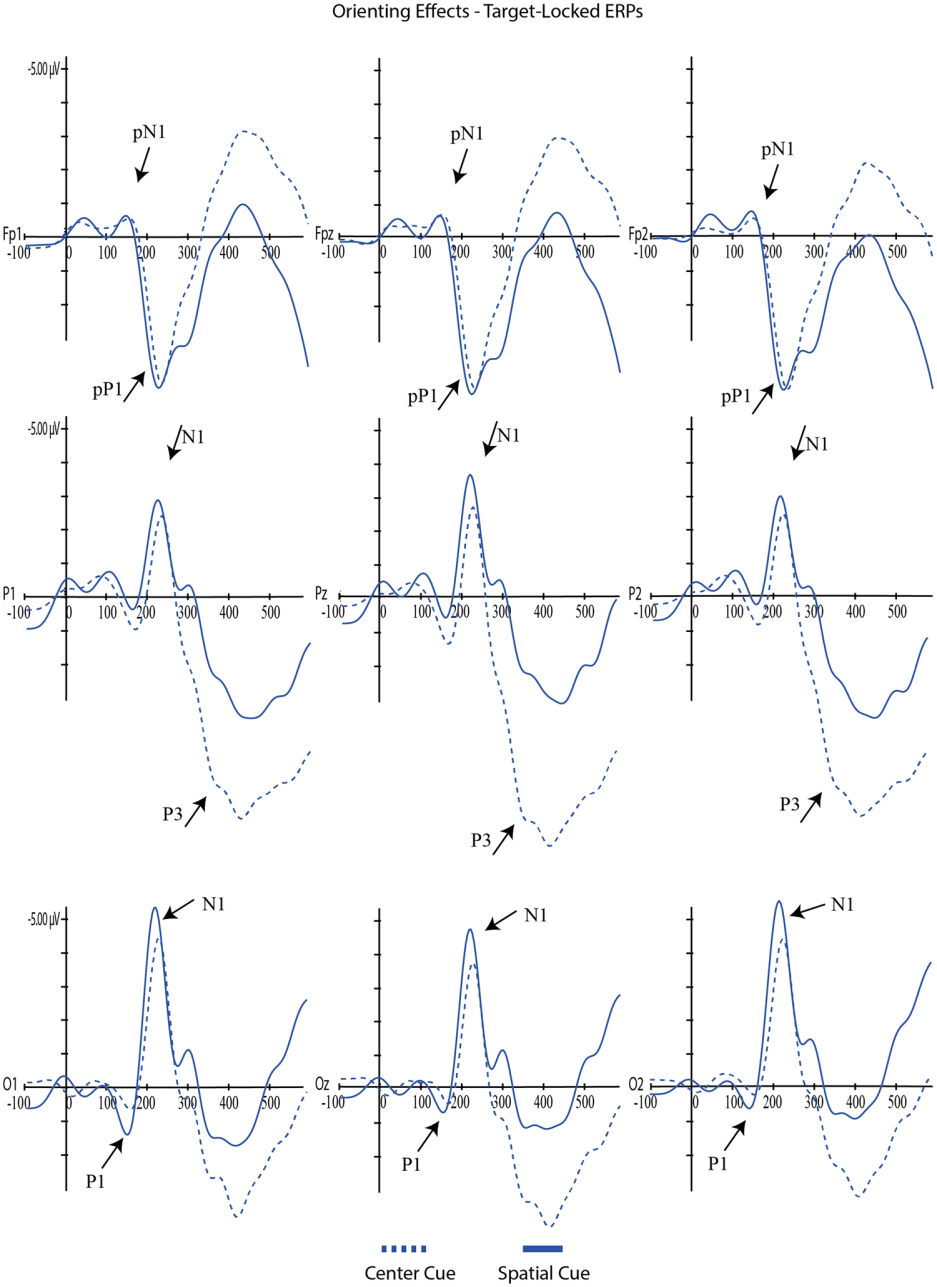
Grand averages for target locked ERP components – orienting effects.

**Figure 8:**
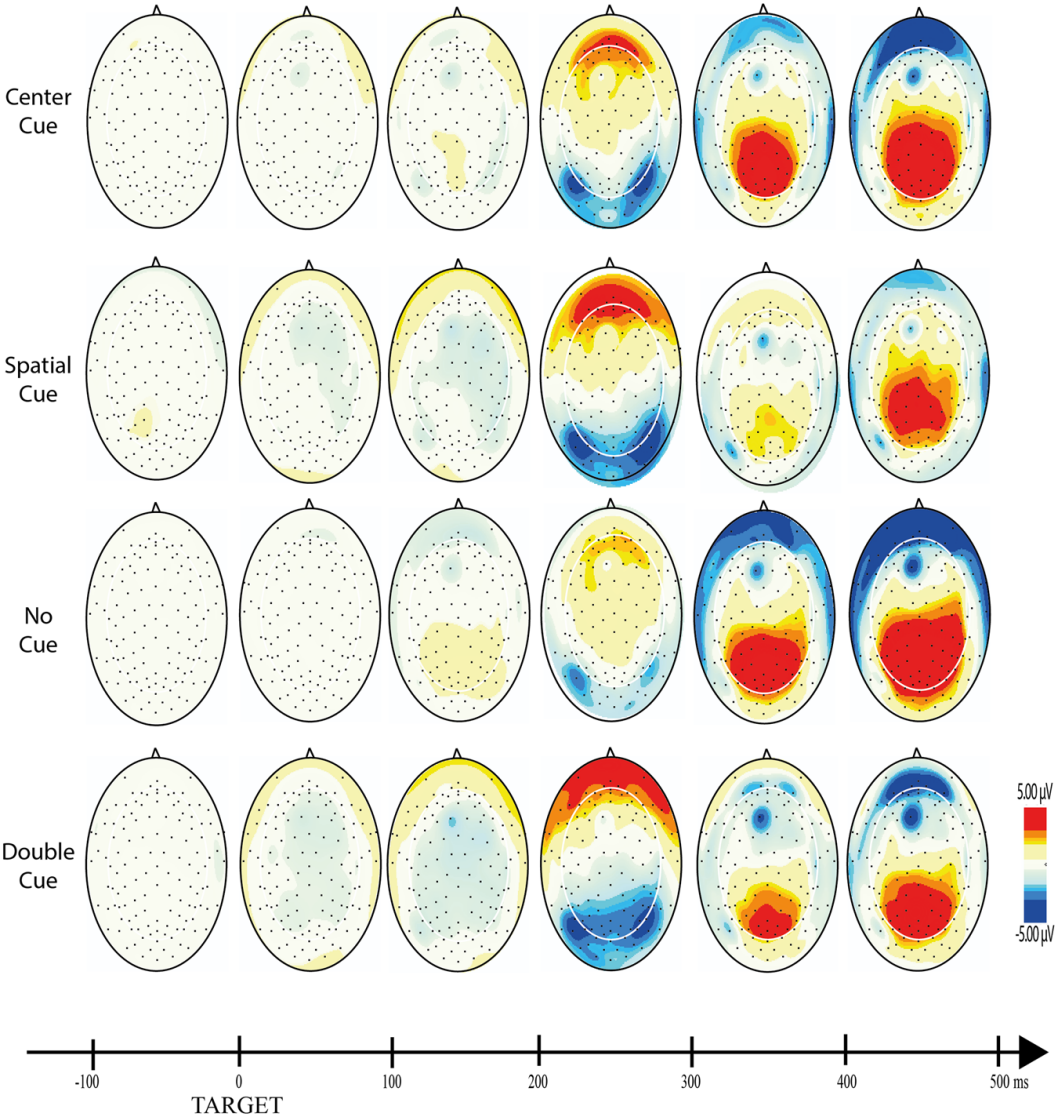
Topographical maps for target locked ERP components.

For each component, mixed ANOVAs were used with adaptive mean amplitude (µV) at the selected electrodes as the dependent variable, type of cue as within-subjects factor (double cue, center cue, spatial cue – for cue locked components; double cue, center cue, spatial cue, no cue – for target locked components) and mind-wandering (MW, FA) as between-subjects factor. For N1 and P3 scalp region (occipital, parietal) was also analyzed as within-factor variable. In the specific case of P3, type of cue was replaced by target type as within-factor variable. Paired comparisons with Student’s t-tests were used to test the direction of significant main or interaction effects. An alpha level of 5% was adopted for all statistical tests. Figures 4, 5, 6, 7 and 8 present the grand-averaged ERP waveforms and topographical maps for all cue locked (Figs 4 and 5) and target locked components (Figs 6, 7 and 8).

#### P1 Component

Regarding the cue-locked P1, no significant effects were found for either mind-wandering [*F*(1,31) = 2.94, *p* = 0.10, ηp^2^ = 0.09] nor for the interaction cue*mind-wandering [*F*(2,62) = 0.14, *p* = 0.87, ηp^2^ = 0.005]. However, a significant main effect was found for cue condition [*F*(2,62) = 14.95, *p* = 0.001, ηp^2^ = 0.33]. Paired-comparisons between double, center and spatial cues showed significant differences between center and spatial cue [*t*(32) = −4.26, *p* < 0.001], as well as between center and double cue [*t*(32) = 2.69, *p* = 0.01] but not between the double and spatial cue [*t*(32) = −1.92, *p* = 0.07]. As illustrated in Fig. 4, both double and the spatial cues resulted in larger P1 amplitudes compared to the center cue.

For the target-locked P1, again there were no significant effects either for mind-wandering [*F*(1,31) = 2.21, *p* = 0.15, ηp^2^ = 0.07] nor for the interaction cue*mind-wandering [*F*(3,93) = 0.14, *p* = 0.94, ηp^2^ = 0.01]; however there was a significant main-effect for cue condition [*F*(3,93) = 5.88, *p* = 0.001, ηp^2^ = 0.16]. As illustrated in Fig. 6, paired-comparisons revealed a significant alerting effect [no cue vs double cue; *t*(32) = 4.04, *p* < 0.001] with increased P1 amplitude for the no cue condition. No significant orienting effects were found for target-locked P1 [center vs spatial cue; *t*(32) = −0.82, *p* = 0.42] (see Fig. 7).

#### pN1 Component

The analysis for cue-locked pN1 component showed no significant effects for mind-wandering [*F*(1,31) = 0.7, *p* = 0.41, ηp^2^ = 0.02] or cue*mind-wandering interaction [*F*(3,93) = 1.7, *p* = 0.17, ηp^2^ = 0.05]. There was a significant main effect for the cue condition [*F*(2,62) = 12.92, *p* = < 0.001, ηp^2^ = 0.29]. Paired comparisons showed significant differences between center - double cue [*t*(32) = 4.5, *p* < 0.001] and center-spatial cues [*t*(32) = 3.57, *p* = 0.001]. Both double and the spatial cues resulted in larger pN1 amplitudes compared to the center cue.

A similar result was found for the target-locked pN1 component, with a significant main effect for *cue* [*F*(3,93) = 3.06, *p* = 0.03, ηp^2^ = 0.09] but no effects for mind-wandering [*F*(1,31) = 1.77, *p* = 0.19, ηp^2^ = 0.05] or cue*mind-wandering interaction [*F*(3,93) = 0.87, *p* = 0.46, ηp^2^ = 0.03]. Paired comparisons showed significant differences between no cue - double cue [*t*(32) = 2.44, *p* = 0.02] and no cue - spatial cue [*t*(32) = −2.28, *p* = 0.03]. As shown in Fig. 6, the no cue condition resulted in a larger pN1 when compared with double and spatial cues.

#### N1 Component

For the cue-locked N1, significant effects were found for cue [*F*(2,62) = 32.34, *p* < 0.001, ηp^2^ = 0.51], scalp [*F*(1,31) = 28.67, *p* < 0.001, ηp^2^ = 0.48] and cue*scalp region interaction [*F*(2,62) = 8.1, *p* = 0.001, ηp^2^ = 0.51], but without significant effects for mind-wandering [*F*(1,31) = 0.51, *p* = 0.48, ηp^2^ = 0.02], cue*mind-wandering [*F*(2,62) = 0.10, *p* = 0.90, ηp^2^ = 0.003], scalp region*mind-wandering [*F*(1,31) = 0.01, *p* = 0.97, ηp^2^ < 0.001] nor for cue*scalp region*mind-wandering [*F*(2,62) = 0.62, *p* = 0.54, ηp^2^ = 0.02]. Paired-comparisons confirmed significant larger N1 amplitude for the double cue when compared with center and spatial cues in parietal [double vs. center, *t*(32) = 5.51, p < 0.001; double vs. spatial, *t*(32) = −8,9, p < 0.001] and occipital regions [double vs. center, *t*(32) = 5.84, p < 0.001; double vs. spatial, *t*(32) = −9,9, p < 0.001].

The analysis of target-locked N1 revealed again significant main-effects for cue condition [*F*(3,93) = 29.87, *p* < 0.001, ηp^2^ = 0.49] and scalp [*F*(1,31) = 8.89, *p* = 0.006, ηp^2^ = 0.22], as well as the interaction between cue*scalp [*F*(3,93) = 8.69, p < 0.001, ηp^2^ = 0.22]. No significant main effects were found for mind-wandering [*F*(1,31) = 0.147, *p* = 0.704, ηp^2^ = 0.005], and interactions mind-wandering*cue [*F*(3,93) = 2.15, *p* = 0.99, ηp^2^ = 0.03], mind-wandering*scalp region [*F*(1,31) = 0.002, *p* = 0.96, ηp^2^ < 0.001], mind-wandering*cue*scalp region [*F*(3,93) = 0.48, *p* = 0.70, ηp^2^ = 0.2]. Table 1 shows the results of paired-comparisons regarding the orienting (no cue vs. double cue targets) and alerting effects (center vs. spatial cues targets) for parietal and occipital regions. N1 adaptive mean amplitudes were larger for spatial and double cues as compared to center (orienting effect) and no cue (alerting effect).

**Table 1:** N1 alerting and orienting effects for each scalp region (Values are described as mean ± SD).

**Orienting Effects**			
	Center cue	Spatial cue	*t*, *p*
Occipital	−2.71 ± 2.27	−3.45 ± 2.08	2.83, 0.01
Parietal	−2.05 ± 2.19	−2.82 ± 1.93	2.76, 0.01
*t, p*	−3.09, 0.004	−2.42, 0.02	
**Alerting Effects**			
	**No cue**	**Double cue**	***t***, ***p***
Occipital	−1.81 ± 2.33	−3.63 ± 2.38	5.33, <0.001
Parietal	−0.83 ± 1.99	−3.61 ± 2.25	9.89, <0.001
*t, p*	−5.40, <0.001	−0.013, 0.90	

#### pP1 Component

For cue-locked pP1 significant main effects were found for the *cue* condition [*F*(2,62) = 15.99, *p* = < 0.001, ηp^2^ = 0.34], but not for mind-wandering [*F*(1,31) = 0.01, *p* = 0.91, ηp^2^ = 0.05] or cue*mind-wandering interaction [*F*(2,62) = 2.57, *p* = 0.09, ηp^2^ = 0.08]. Paired comparisons showed pP1 larger adaptive mean amplitudes for the double condition when compared with center cue [*t*(32) = −5.65, *p* < 0.001] and spatial cue [*t*(32) = 5.42, *p* < 0.001].

Concerning target-locked pP1, again significant effects were found for cue [*F*(3,93) = 7.94, *p* = < 0.001, ηp^2^ = 0.20], but not for mind-wandering [*F*(1,31) = 1.97, *p* = 0.17, ηp^2^ = 0.06] nor cue*mind-wandering interaction [*F*(3,93) = 0.56, *p* = 0.64, ηp^2^ = 0.02]. Post-hoc comparison showed smaller adaptive mean amplitudes for the no cue when compared with the all other cue conditions [no cue and center cue, *t*(32) = 3.9, *p* < 0.001; no cue and double cue, *t*(32) = 3.17, *p* = 0.003; no cue and spatial cue, *t*(32) = −4.1, *p* < 0.001].

#### pN Component

For cue-locked pN, significant main effects were found for the cue [*F*(2,62) = 10.22, *p* < 0.001, ηp^2^ = 0.25], but not for mind-wandering [*F*(1,31) = 2.4, *p* = 0.13, ηp^2^ = 0.07] nor for the interaction cue*mind-wandering [*F*(2,62) = 0.52, *p* = 0.60, ηp^2^ = 0.02]. Paired-comparisons revealed significant larger adaptive mean amplitude for the spatial cue when compared with all the other cues [double cue, *t*(32) = 4, *p* < 0.001; center cue, *t*(32) = 3.4, *p* = 0.002].

#### P3 Component

Regarding the target-locked P3, significant main-effects were found for the target condition [*F*(2,62) = 25.31, *p* < 0.001, ηp^2^ = 0.45], scalp region [*F*(1,31) = 77.30, *p* < 0.001, ηp^2^ = 0.71] as well as the interaction target*scalp region [*F*(2,62) = 8.53, p < 0.001, ηp^2^ = 0.22]. Again, no significant main effects were found for of mind-wandering [*F*(1,31) = 0.38, *p* = 0.54, ηp^2^ = 0.01], and interactions mind-wandering*target [*F*(2,62) = 0.25, *p* = 0.77, ηp^2^ = 0.008], mind-wandering*scalp region [*F*(1,31) = 3.19, *p* = 0.08, ηp^2^ = 0.09], mind-wandering*target*scalp region [*F*(2,62) = 1.56, *p* = 0.22, ηp^2^ = 0.05].

Paired-comparisons were performed between congruent, incongruent and neutral targets for parietal and occipital regions. Table 2 shows that P3 adaptive mean amplitudes were significantly larger for congruent and neutral as compared to incongruent targets in the parietal and occipital electrodes (conflict effect). We found larger P300 components for the parietal as compared to the occipital electrodes for congruent (*t32* = −7.98, *p* < 0.001), incongruent (*t32* = −7.59, *p* < 0.001) and neutral (*t32* = −8.23, *p* < 0.001).

**Table 2:** P3 conflict effects for each scalp region.

Comparisons	Parietal	*t*	*p*	Occipital	*t*	*p*
Congruent Incongruent	5.73 ± 2.65 4.16 ± 2.97	5.95	<0.001	3.55 ± 2.05 2.14 ± 2.73	5.09	<0.001
Congruent Neutral	5.73 ± 2.65 6.13 ± 2.59	−1.98	0.06	3.55 ± 2.05 3.56 ± 2.38	−0.01	0.99
Incongruent Neutral	4.16 ± 2.97 6.13 ± 2.59	−7.17	<0.001	2.14 ± 2.73 3.56 ± 2.38	−4.20	<0.001

## Discussion

The present study investigates the modulatory effects of MW thoughts on the ERP correlates of attention networks (i.e., alerting, orienting and conflict). As expected, ANT’s behavioral results showed evidence of significant alerting, orienting and conflict effects. Participants were slower and less accurate when responding to incongruent than to congruent targets (conflict effect) and benefit from the presentation of double (alerting effect) and spatial cues (orienting effect). These findings remain true irrespective of the MW condition. Participants from the MW group performed similarly to the FA group, showing the same attention effects (alerting, orienting, and conflict). This data is consistent with previous findings showing the lack of interference of MW on ANT’s overall performance or any of the attention networks^10,16^. A previous study found a negative correlation between MW with somatosensory awareness content and the alerting network, however this was not generalized across all the study population^16^. This MW content is probably more associated with “external distractions” than to pure MW, a category that has been found to interfere with ANT accuracy^10^.

The central aim of the present study was, however, to explore if MW impacts the ERP correlates of alert, orienting and conflict attention tasks. Overall, the ERP’s analyzed were discriminative of the three attentional effects (alerting, orienting, conflict) irrespective of the MW condition.

For cue-locked P1 we found an amplitude increase with the information power of the cue (significant increases from center cue, to double cue and spatial cue, and approaching significance between double and spatial cues). P1 is considered an early marker of visual attention generated in the extrastriate region of the visual cortex, showing an enhanced amplitude as the informative power of the cue increases^44,45^. When we moved to a target locked P1 we found a decrease in P1 for alerting along with a lack of an orientation effect. This is consistent with Williams^32^ and colleagues study, showing an increased amplitude in the no cue condition compared with the double cue condition. However, Galvao-Carmona and colleagues reported the opposite finding of an increased P1 orienting effect and no significant alerting effects. Williams^32^ considered that the discrepancy in these findings may be associated with the different scalp locations where the effects were found: averaged at P3, Pz, P4, O1, Oz, O2 in Williams study and PO5 and PO6 in Galvao-Carmona study. Our findings support this explanation as we analyzed the P1 component at O1, Oz and O2. Furthermore, it seems that the spatial distribution of the P1 at the occipital sites reflects the absence of an alerting cue by an enhanced no-cued target P1. It is known that the delay between cue and target also impacts the amplitude of the P1 component^46,47^. Our ANT protocol was similar to the one used by Williams (100 ms for the cue followed by a 400 ms interval before the target) while Galvao-Carmona used a larger delay between the cue and the target (150 ms for the cue followed by 1000 ms before the target). Therefore, P1 seems to be a specific cue elicited marker for the amount of the cue information, dependent also of the cue-target delay as well as spatial distribution. Summing up, both Williams and our current experiment suggest that the lack of cue information enhances the alert at the early stages of visual processing as reflected by an enhanced P1 at occipital electrodes.

N1 has been described as an early visual attention component, generated at both parietal and occipital regions. The present study confirmed that target locked N1 is a good marker for both the alerting and the orienting effects, with increased amplitudes for the double cue (alerting effect) and spatial cues (orientation effect) both at occipital and parietal regions (even though more evident in the occipital region). Similar findings were reported in previous studies^32,33^. The present results are in line with Neuhaus^30^ and colleagues showing an increase in N1 amplitudes for the alerting and orienting conditions as well as significant interaction with electrode site (alerting effects mostly observed at parietal electrodes and orienting effects at occipital and parieto-occipital electrodes). Consistent with our data, the author found that increases in N1 amplitude were particularly evident after alerting cues.

Similar to the studies reported above, N1 amplitude increased in the following order: no cue, center cue, spatial, double cue. This finding confirms that N1 is more sensitive to the alerting effects and that the orientation effect found may result from the relative interdependence of the networks as measured by the ANT (e.g., orienting cues have also alerting effects)^42,48^.

As anticipated, prefrontal activity ERP markers were discriminative for distinct attentional effects but, once again, irrespective of the MW condition. Therefore, there was no evidence for compensatory prefrontal activity as expressed in pN1, pP1 or pN. However, larger pN1 and pP1 amplitudes were associated with increments in cue informative power (double and spatial cues when compared to the center cue) for the cue-locked interval. Moreover, alerting, but no orienting effects, were present in pN1(smaller amplitude in the double cue when compared with no cue condition) and pP1 (smaller amplitude in the no cue when compared with double cue condition). These findings bring additional confirmation for the hypothesis that these markers of prefrontal activity reflect early perceptual processes of stimulus evaluation. Previous studies have suggested that these ERPs are detected in stimulus locked but not response locked paradigms^37^.

Similar findings were present in the cue-locked pN with an increased negativity, but in this case only in response to the spatial cue. The pN is a slow negative potential (350–500 ms after cue onset) with a medial frontal scalp distribution and has been thought to reflect proactive inhibitory control^49^. This ERP is dependent on a bilateral activation of the *pars opercularis*, and occurs of weather the reaction following stimulus detection is inhibitory or not^50^. The increased negativity in response to the spatial cue suggests an early sensitivity (between the warning cue and the target) to perceptual interpretation, probably due to proactive inhibitory processes involved in orienting attention.

Reduced P3 amplitude was found for the incongruent targets when compared with the congruent and the neutral conditions. These P3 effects for all targets were more clear in parietal regions. This confirms the interfering effects of incongruent targets in attention performance as evidenced by a decreased of accuracy and increased in RT for incongruent targets. Other studies have found similar reduced P3 amplitudes at parietal sites for incongruent targets^30–33^. Our finding of a P3 reduction for the incongruent target when compared with congruent and neutral targets, but not between the congruent and neutral targets, suggests that the P3 effects seem to be specific for situations of attention conflict. Neuroimaging studies showed an increased activation of the anterior cingulate (a region involved in conflict resolution) for incongruent tasks^22^. The anterior cingulate has been identified as a core source for the P3^51^.

Finally, consistent with the lacking of MW effects on ANT performance, there were no differences between the MW and FA groups in pN, P1, and N1 ERP’s for orienting and alerting cues and P3 for incongruent targets, suggesting that MW is still compatible with effective early alerting and orienting visual attention processes and resolution of attention conflict.

The aim of the present study was to test if the lack of ANT task costs in MW could be explained by an increased cortical demand illustrated in the modulation of cortical activity. Contrary to previous studies, we found that alert, orienting and conflict attention ERP’s markers were independent of MW condition. However, none of those studies used the ANT to assess attention. For example, modulation of P3^34^, P2^35^ and P1^36^, was evident when individuals mind wander in response to non-targets or targets in vigilant tasks (SART, 0-back vigilant), or passive odd-ball stimuli. More important, in these studies, MW was broadly defined, including both task-related and internal distractors (e.g., losing track of their thoughts during a breath count task^35^; thinking about anything unrelated to the task^36^; “tuned out” – consciously away from the task or “zoned out” – unconsciously off-task^34^). It is possible that the modulation of cortical activity reported in these studies, is associated with the presence of external and task distractors rather than MW *per se*. Interesting to note that, contrary to our study, previous research has reported cost effects for off-task thoughts. For example, Baird and colleagues found significant increases in RT in responding to non-frequent targets in a 0-back vigilance tasks when preceded by off-task thoughts^36^.

The present findings bring further evidence to the assumption that the level of processing required for ANT performance is compatible with mind wandering. As suggested by some authors, individuals with good executive resources are able to mind-wander without significant impact on their attention in non-demanding tasks at the behavioral level^28^. The present study shows that this may be also true at the neurophysiological level. That is, no evidence was found for additional cortical demand in MW for identical performance on the ANT. It is possible that, with increased task demand, additional cortical recruitment would be required even before response costs are evident. Future studies should test this possibility by manipulating ANT task-related demand.

In the current ANT paradigm a fixed 400 ms cue-target interval was used, precluding the analysis of different Stimulus Onset Asynchrony’s **(SOA’s)** in ANT performance and ERP correlates. For example, studies show that at the behavioral level, the classical cue-target effect is evident for intermediate and long SOAs, whereas for short SOAs, there was a time cost associated with valid cues^52^. Additionally, several studies confirm the influence of SOA’s on ERPs responses. Indeed, ERPs seem to be depend on different SOA interval^53^, order^54^ and experimental conditions^55^. Therefore it would be interesting to test the modulation of MW in ANT at the behavioral and neurophysiological levels controlling for different SOA’s.

Despite the consistency between the behavioral and ERP data, the present results should be interpreted in light of some methodological limitations. One of the more complex issues in this research domain is finding a reliable way for assessing MW, namely the choice between the use of real time or retrospective strategies^14^. Real time probes are more effective in providing a reliable account of interfering thoughts. In the case of the ANT, in which different trials within each block are measuring different network effects, an effective real-time probe would be required for each trial. However, this would interrupt the sequence of trials within each block, interfering with the ANT paradigm. In previous studies we opted for a thought probe encompassing the whole block and not only the preceding trial^10^. However, this strategy would be prone to recency effects, with participants eventually biasing their reports towards the immediate trial preceding the thought probe. This is the main reason underlying the choice for the retrospective offline report on the content of interfering thoughts in this study. This strategy has the advantage of not interfering with the attention task, being also more resistant to recency effects. Nevertheless, we cannot be sure about the reliability of participants’ retrospective offline reports. Some authors recommended the priming of MW or FA conditions^56^. For example, a recent study showed that the stimulation of the left dorsolateral prefrontal cortex with transcranial direct current stimulation increased MW. Consistent with our data, rather than having a negative impact, the increase in MW induced a small attention improvement.

In conclusion, at the behavioral level, no significant interfering MW effects were found in ANT performance (reaction time, accuracy and network effects). Consistent with these findings, ERP markers associated with distinct attentional effects (alert, orienting, conflict) were not modulated by the MW condition. Future studies should try to replicate these results with larger samples, controlling for task-related demands and SOA’s while experimentally priming MW/FA conditions.
